# Estrogen regulates T helper 17 phenotype and localization in experimental autoimmune arthritis

**DOI:** 10.1186/s13075-015-0548-y

**Published:** 2015-02-13

**Authors:** Annica Andersson, Alexandra Stubelius, Merja Nurkkala Karlsson, Cecilia Engdahl, Malin Erlandsson, Louise Grahnemo, Marie K Lagerquist, Ulrika Islander

**Affiliations:** Department of Rheumatology and Inflammation Research, Centre for Bone and Arthritis Research, The Sahlgrenska Academy, University of Gothenburg, Box 480, 405 30 Gothenburg, Sweden; Department of Internal Medicine and Clinical Nutrition, Centre for Bone and Arthritis Research, The Sahlgrenska Academy, University of Gothenburg, Vita Stråket 11, 413 45 Gothenburg, Sweden

## Abstract

**Introduction:**

The incidence and progression of many autoimmune diseases are sex-biased, which might be explained by the immunomodulating properties of endocrine hormones. Treatment with estradiol potently inhibits experimental autoimmune arthritis. Interleukin-17-producing T helper cells (Th17) are key players in several autoimmune diseases, particularly in rheumatoid arthritis. The aim of this study was to investigate the effects of estrogen on Th17 cells in experimental arthritis.

**Methods:**

Ovariectomized DBA/1 mice treated with 17β-estradiol (E2) or placebo were subjected to collagen-induced arthritis (CIA), and arthritis development was assessed. Th17 cells in joints and lymph nodes were studied by flow cytometry. Lymph node Th17 cells were also examined in ovariectomized estrogen receptor α–knockout mice (ERα^−/−^) and wild-type littermates, treated with E2 or placebo and subjected to antigen-induced arthritis.

**Results:**

E2-treated mice with established CIA showed reduced severity of arthritis and fewer Th17 cells in joints compared with controls. Interestingly, E2-treated mice displayed increased Th17 cells in lymph nodes during the early phase of the disease, dependent on ERα. E2 increased the expression of C-C chemokine receptor 6 (CCR6) on lymph node Th17 cells as well as the expression of the corresponding C-C chemokine ligand 20 (CCL20) within lymph nodes.

**Conclusions:**

This is the first study in which the effects of E2 on Th17 cells have been characterized in experimental autoimmune arthritis. We report that E2 treatment results in an increase of Th17 cells in lymph nodes during the early phase of arthritis development, but leads to a decrease of Th17 in joints during established arthritis. Our data suggest that this may be caused by interference with the CCR6-CCL20 pathway, which is important for Th17 cell migration. This study contributes to the understanding of the role of estrogen in the development of autoimmune arthritis and opens up new fields for research concerning the sex bias in autoimmune disease.

**Electronic supplementary material:**

The online version of this article (doi:10.1186/s13075-015-0548-y) contains supplementary material, which is available to authorized users.

## Introduction

Sex influences susceptibility to autoimmune diseases such as rheumatoid arthritis (RA), for which the female-to-male ratio is 3:1. The peak incidence of RA in women coincides with the time of menopause, when estrogen levels rapidly drop, connecting sex hormones to disease etiology [1]. In contrast, men have rather continuous levels of estrogen throughout their adult lives, and estrogen levels are lower in postmenopausal women than in men of corresponding age [2]. During pregnancy, when sex hormone levels rise, up to 75% of RA patients experience relief of disease symptoms [3]. In a well-established experimental model of RA, collagen-induced arthritis (CIA), it has repeatedly been shown that estrogen ameliorates disease development [4,5]. Regarding human RA, some studies indicate that hormone replacement therapy including estradiol might be beneficial; however, the results of studies in this field are inconsistent. In one long-term study, researchers reported improved disease activity scores and increased bone mineral density after hormone replacement therapy [6]. Secondary osteoporosis is common in RA patients; about 50% of postmenopausal women with RA have a diagnosis of osteoporosis [7]. In contrast to RA, estrogen aggravates systemic lupus erythematosus [8]. Indeed, estrogen is a potent immunomodulatory agent and can exert stimulatory as well as regulatory effects on the immune system, such as enhancing B cell antibody production, reducing B and T lymphopoiesis and inhibiting T cell-dependent inflammation [9-12].

The cytokine interleukin (IL)-17A (referred to as IL-17) is produced mainly by T helper 17 cells (Th17) and constitutes the driving force in several autoimmune diseases. In RA, Th17 cell frequency and level of synovial fluid IL-17 strongly correlate with disease activity [13]. Phase II clinical trials on RA patients receiving anti-IL-17A treatment resulted in significantly decreased disease activity scores [14,15]. IL-17 augments joint inflammation by stimulating synovial fibroblasts to produce CXCL8 (IL-8), thereby attracting neutrophils to the joints [16,17]. Moreover, IL-17 plays a role in inflammation-induced bone loss by stimulating osteoclastogenesis [18]. Migration of Th17 cells to the site of inflammation is mainly orchestrated by the interaction of C-C chemokine ligand 20 (CCL20) with C-C chemokine receptor 6 (CCR6) that is expressed on the Th17 cell [19].

Effects of estrogen on Th17 cells have mostly been studied in the context of experimental multiple sclerosis—experimental autoimmune encephalomyelitis (EAE)—another Th17-driven disease where estrogen is protective. Estrogen decreases production of IL-17 in EAE and inhibits Th17 differentiation and disease progression, dependent on estrogen receptor α (ERα) in T cells [20,21]. However, effects of estrogen on the Th17 cell population in arthritis have been scarcely studied, and are limited to studies on IL-17 production. We have recently shown that estrogen decreases splenic IL-17 production *in vitro* in antigen-induced arthritis (AIA) in an ERα-dependent manner [22]. Furthermore, the estrogen metabolite 2-methoxyestradiol decreased IL-17 mRNA in arthritic joints in collagen-antibody induced arthritis [23].

In this study, we thoroughly characterized estrogenic effects on Th17 cell phenotype and localization in experimental arthritis. We demonstrate that estrogen regulates localization of Th17 cells during development of CIA, resulting in increased Th17 in lymph nodes (LNs) but decreased Th17 in joints. In addition, the E2-mediated increase in Th17 cells in LNs is dependent on ERα. Moreover, estrogen increases CCR6 expression on LN Th17 cells and enhances production of CCL20 within LNs, possibly resulting in Th17 cell accumulation in the LNs and reduced migration of Th17 cells to joints. Subsequently, IL-17-mediated destruction of joints is inhibited. Our study increases the understanding of how estrogen regulates the immune system in autoimmune diseases.

## Methods

### Animals

The study was approved by the regional ethical review board in Gothenburg, Sweden. Female DBA/1 mice (Taconic Europe A/S, Ry, Denmark), total ERα-inactivated mice (ERα^−/−^) and wild-type (WT) littermates were electronically tagged and kept in groups of five to ten animals in each cage under standard environmental conditions. The mice were fed standard laboratory chow and tap water *ad libitum*. ERα^−/−^ mice have a deletion in exon 3 of the ERα gene and do not express any of the isoforms of the ERα protein. ERα^−/−^ mice and WT (ERα^+/+^) littermates were inbred C57BL/6 mice. They were generated by breeding male ERα^+/−^ with female ERα^+/−^ mice, as previously described [24].

### Ovariectomy

All mice were ovariectomized (OVX) initially. At 8 to 10 weeks of age, ovaries were removed through a midline incision of the skin followed by flank incisions of the peritoneum, and the skin was closed with metallic clips. Surgery was performed with the mice under isoflurane-induced anesthesia (Baxter Healthcare, Chicago, IL, USA), and carprofen (Rimadyl; Orion Pharma Animal Health, Sollentuna, Sweden) was used as a postoperative analgesic.

### Arthritis models

#### Collagen-induced arthritis

**Figure 1:**
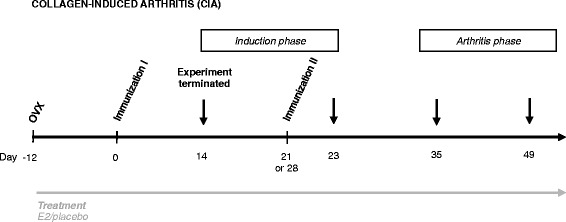
**Experimental setup for collagen-induced arthritis.** Female DBA/1 mice were ovariectomized (OVX) at 8 to 10 weeks of age and simultaneously received an implanted slow-release pellet containing 17β-estradiol (E2; 0.83 μg/day) or corresponding placebo. After 12 days of rest, they were immunized subcutaneously at the base of the tail with collagen type II and Freund’s adjuvant (day 0), which was repeated at day 21 or 28. Experiments were terminated at days 14, 23, 35 and 49 after the first immunization. Days 14 and 23 were considered as time points in the (asymptomatic) induction phase of collagen-induced arthritis (CIA), whereas days 35 and 49 were defined as established (symptomatic) CIA.

The CIA experimental protocol is depicted in Figure 1. 12 days after ovariectomy, DBA/1 mice were immunized with chicken collagen type II (100 μg/mouse; Sigma-Aldrich, St Louis, MO, USA) in Freund’s incomplete adjuvant (Sigma-Aldrich) supplemented with *Mycobacterium tuberculosis* H37 RA (50 μg/mouse; BD, Franklin Lakes, NJ, USA) (day 0), as described elsewhere [25]. Immunization was repeated after 21 or 28 days, without mycobacteria. Arthritis development was monitored and scored by examination of mice every second or third day in a blinded manner with respect to treatment groups. Arthritis severity was assessed by using scores (0 to 3) for each paw, giving a maximum of 12 points for each mouse, determined as follows: 1 = swelling or erythema in one joint, 2 = swelling or erythema in two joints, 3 = severe swelling or erythema of more than two joints, or ankylosis of the entire paw. Experiments were terminated at several time points after the first immunization (Figure 1).

#### Antigen-induced arthritis

Two weeks after ovariectomy, ERα^−/−^ mice and WT littermates were subjected to AIA by immunization at day 0 with methylated bovine serum albumin (mBSA; in phosphate-buffered saline (PBS), 0.2 mg/mouse; Sigma-Aldrich) emulsified in Freund’s complete adjuvant (Sigma-Aldrich) injected at the base of the tail, followed by intraarticular injection of mBSA (0.3 mg/mouse) into one knee at day 7. The AIA experiment was terminated day 14. AIA is described in detail elsewhere [22].

### Treatment

Mice were administered subcutaneous slow-release implants with 17β-estradiol (E2; Innovative Research of America, Sarasota, FL, USA) or placebo at the time point of OVX, resulting in daily doses of 0.83 μg of E2 per mouse. In one CIA experiment, mice received only 3 days of E2 treatment, administered by subcutaneous injections during days 20 to 22, using 17β-estradiol-3-benzoate (1 μg/mouse/day: Sigma-Aldrich) in inert oil (Miglyol 812; Recip, Årsta, Sweden), and control mice received oil only. As described earlier, mice treated with estradiol in doses similar to those used herein obtain serum estradiol of 10 to 25 pg/ml, which in healthy mice corresponds to low diestrus levels [26]. Successful OVX and estrogen treatment was confirmed by weighing uteri at termination (data not shown).

### Tissue collection and histological examination

At termination, mice were anesthetized with ketamine (Ketalar; Pfizer AB, Täby, Sweden) and medetomidine (Domitor; Orion Pharma Animal Health), bled, and killed by cervical dislocation. Sera were stored at −20°C until use. Paws from CIA mice were either placed in RPMI 1640 for isolation of joint cells or placed in 4% formaldehyde, decalcified, embedded in paraffin, sectioned and stained with hematoxylin and eosin. Synovitis and erosions were separately scored from 0 to 3: 0 = normal appearance, 1 = mild, 2 = moderate or 3 = severe synovitis/erosion of cartilage and bone. A histopathological index was constructed by adding the scores from the evaluated joints in each animal. LNs draining the joints in CIA (subiliac, popliteal, sciatic, proper and accessory axillary) and in AIA (subiliac, popliteal) [27] were pooled and stored in PBS until cell preparation, or snap-frozen, or kept in RNA*later* (Qiagen, Hilden, Germany), then stored in −70°C for mRNA analysis.

### Preparation of cells and flow cytometric analysis

To isolate cells from the joints of arthritic mice, paws were stripped from skin and digested with collagenase IV (Sigma-Aldrich) and subsequently passed through a 70-μm cell strainer. LNs were mashed through a 70-μm cell strainer and washed in PBS. Cells were counted in an automated counter (Sysmex, Noderstedt, Germany). For analysis of intracellular cytokines, cells were suspended in complete medium (phenol red-free RPMI 1640 (PAA Laboratories, Pasching, Austria) supplemented with 10% dextran-coated charcoal hormone-stripped fetal calf serum (Sigma-Aldrich), 1% 2-mercaptoethanol (Sigma-Aldrich) and 1% penicillin-streptomycin-l-glutamine solution (Sigma-Aldrich)), and then stimulated with phorbol 12-myristate 13-acetate (PMA) (50 ng/ml; Sigma-Aldrich), ionomycin (1 μg/ml: Sigma-Aldrich) and GolgiPlug (BD) for 4 hours at 37°C and 5% CO_2_. Fluorochrome-conjugated anti-mouse antibodies were used for intracellular staining of IL-17A (eBioscience, Vienna, Austria), interferon γ (IFNγ) (BD) and Foxp3 (eBioscience) in permeabilization buffer (eBioscience) and for extracellular staining of CD4, CD11b and CD25 (BD); CCR2 and S1PR1/EDG-1 (R&D Systems, Minneapolis, MN, USA); and CCR6, CCR7, F4/80 and Gr-1 (BioLegend, San Diego, CA, USA). All analyses started with gates on singlet cells; thereafter, Th17 (CD4^+^IL-17^+^), Th1 (CD4^+^IFNγ^+^), and regulatory T cells (Treg; CD4^+^Foxp3^+^CD25^+^) were gated on lymphocytes, and neutrophils (CD11b^+^F4/80^−^Gr-1^hi^) were gated on live cells. For each individual, absolute numbers of cell populations were obtained by multiplying the frequency of the cell population obtained by flow cytometry by the total cellularity of either one subiliac LN or the four paws. Samples were run on a BD FACSCanto II flow cytometer, and data were processed using FlowJo 8.8.6 software (Tree Star, Ashland, OR, USA).

### IL-17A ELISPOT

The ELISPOT assay was performed according to manufacturer’s instructions (eBioscience). Added to each well were 0.5 to 1 × 10^5^ joint cells, or all migrated LN cells from the chemotaxis assay, followed by stimulation with PMA and ionomycin. After incubation at 37°C and 5% CO_2_ for 40 to 42 hours, spots were developed and counted.

### Chemotaxis assay

LN cells (from CIA mice treated with E2 or placebo, day 35) were cultured in a 24-well, 5-μm pore size Transwell system (Corning Costar, Corning, NY, USA). A total of 2 × 10^6^ cells were placed on top of the transwell, and CCL20 (50 ng/ml; R&D Systems), or complete medium only, was added to the lower chamber. After 4 hours of incubation (37°C and 5% CO_2_), migrated cells were put on an IL-17 ELISPOT assay, performed as described above. The percentage CCL20-induced migration of IL-17-producing cells in each treatment group was calculated as follows: number of IL-17 spot-forming cells (SFCs) after migration toward CCL20 minus number of IL-17 SFCs after migration toward medium only (background), divided by number of IL-17 SFCs before chemotaxis assay.

### ELISA

Serum CCL20 quantification was performed using a mouse CCL20/macrophage inflammatory protein 3α DuoSet enzyme-linked immunosorbent assay (ELISA) kit (R&D Systems). Plates (Corning Costar) were coated with anti-CCL20 monoclonal antibody and blocked (1% BSA). After adding samples, detection antibody and streptavidin-horseradish peroxidase, plates were developed with 3,3′,5,5′-tetramethylbenzidine (Sigma-Aldrich), stopped with 1 M H_2_SO_4_ and read at an absorbance of 450 nm, with wavelength correction at 540 nm, on a SpectraMax Plus (Molecular Devices, Sunnyvale, CA, USA). Serum sphingosine-1-phosphate (S1P) levels were determined by using a competitive S1P ELISA kit (Echelon Biosciences, Salt Lake City, UT, USA), according to the manufacturer’s instructions.

### Real-time quantitative PCR

Subiliac LNs were homogenized in TissueLyser II (Qiagen), and total RNA was extracted using the RNeasy Mini kit in QIAcube (Qiagen). RNA content, purity and integrity were determined with NanoDrop ND-1000 (NanoDrop Technologies, Wilmington, DE, USA) and Experion (Bio-Rad Laboratories, Hercules, CA, USA). A high-capacity cDNA reverse transcription kit (Applied Biosystems, Foster City, CA, USA) was used to perform cDNA synthesis. Real-time quantitative PCR was performed at the Genomics Core Facility, University of Gothenburg, using a Biomek FX pipetting robot (Beckman Coulter, Fullerton, CA, USA) and an ABI PRISM 7900 HT instrument (Applied Biosystems). The genes of interest and endogenous controls were investigated with inventoried TaqMan Gene Expression assays (*Ccl20*: Mm01268754_m1, *Ccl2*: Mm00441242_m1, *Ccl12*: Mm01617100_m1, *Ccl19*: Mm00839967_g1, *Ccl21*: Mm03646971_gH, *18 s*: Mm03928990_g1, *B2m*: Mm00437762_m1, *Eef2*: Mm01171435_gH, *Hprt1*: Mm01545399_m1, *Rpl13a*: Mm01612986_gH) and TaqMan Gene Expression Master Mix (Applied Biosystems). mRNA expression analysis was performed with RQ Manager software version 1.2.1 (Applied Biosystems). The *Eef2* and *Hprt1* combination was identified as the optimal normalization gene pair using NormFinder software (Molecular Diagnostic Laboratory, Aarhus University Hospital, Aarhus, Denmark). Gene expression values were calculated using the 2^−ΔΔ*C*T^ relative quantification method, wherein the placebo group was designated the calibrator [28].

### Statistical analysis

Statistical evaluations were performed using IBM SPSS software 20.0.0 (IBM, Armonk, NY, USA) and GraphPad Prism version 6.0b software (GraphPad Software, La Jolla, CA, USA). Student’s *t*-test was used for comparison of two independent groups. Whenever Levene’s test revealed unequal variances between the groups, Welch’s *t*-test was used instead. Logarithmic transformations were used when appropriate to ensure normal distribution of data. Analysis of covariance was used when adjustments for covariates were needed (that is, when two experiments were pooled). The area under the curve for severity of arthritis was calculated by the trapezoidal method. Scoring of severity of arthritis and histopathological index were performed using an ordinal scale requiring non-parametric statistical evaluation, and therefore the Mann–Whitney test was used. A log-rank test was performed to compare the incidence of arthritis between treatment groups. *P*-values <0.05 were considered statistically significant.

## Results

### E2-treated mice have fewer Th17 cells in joints and less severe arthritis than placebo controls

**Figure 2:**
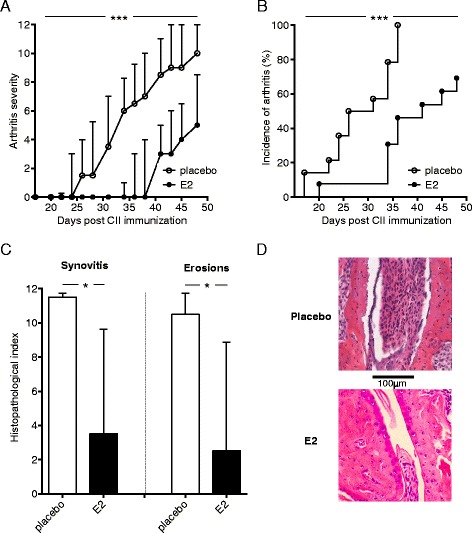
**Effects of estrogen on arthritis development in the collagen-induced arthritis model.** DBA/1 mice were ovariectomized, subjected to collagen-induced arthritis (CIA) and treated with 17β-estradiol (E2; 0.83 μg/day) or placebo. Arthritis development was macroscopically assessed every second or third day by a blinded examiner, yielding a maximum of 12 points per mouse (single experiment with *n* = 14 mice/group). **(A)** Severity of arthritis is expressed as median and interquartile range. The area under the curve was calculated for each treatment group and differences between groups were analyzed by Mann–Whitney test. CII, Collagen type II. **(B)** Incidence of arthritis is presented as Kaplan-Meier curves and analyzed by log-rank test. **(C)** Microscopic synovitis and erosions on bone and cartilage were assessed in paw sections stained with hematoxylin and eosin, from placebo- or E2-treated mice with CIA, terminated on day 49. Data are median with interquartile range, Mann–Whitney test, *n* = 5 or 6 mice/group in a single experiment. Representative sections from each treatment group are shown in **(D)**. Original magnification, ×20. *P <0.05, ***P <0.001.

**Figure 3:**
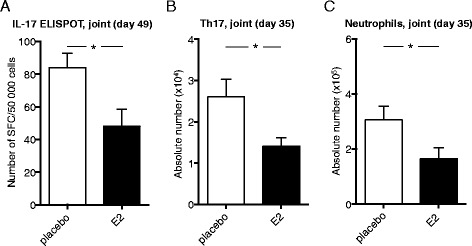
**Effects of estrogen on Th17 cells in joints of mice with established collagen-induced arthritis.** DBA/1 mice were ovariectomized, subjected to collagen-induced arthritis (CIA) and treated with 17β-estradiol (E2; 0.83 μg/day) or placebo. **(A)** Joint cells from mice with CIA terminated day 49 were stimulated in triplicates with phorbol 12-myristate 13-acetate and ionomycin for 40 to 42 hours in interleukin (IL)-17 ELISPOT assay. The number of spot-forming cells (SFCs) was counted in a blinded manner these are shown as SFCs per total number of cells in wells. Total numbers of **(B)** T helper 17 cells (Th17; IL-17^+^CD4^+^) and **(C)** neutrophils (CD11b^+^F4/80^−^Gr-1^hi^) in joints from mice with CIA terminated day 35 were determined by flow cytometry. The absolute number of cells was derived by multiplying the frequency of Th17 cells or neutrophils by the total number of cells acquired from an automated cell counter in a corresponding sample. All data shown are arithmetic mean ± SEM derived from single experiments with *n* = 8 or 9 mice/group. *P <0.05 (Student’s *t*-test).

Female DBA/1 mice underwent OVX to reduce endogenous hormone production. Mice were randomly assigned to treatment with E2 or placebo and subjected to CIA, and the development of arthritis was assessed. E2 dramatically decreased the severity and incidence of arthritis and delayed the onset of disease (Figure 2A,B). Histological examination of paws confirmed the E2-mediated amelioration of arthritis, regarding the presence of both synovitis and erosions on cartilage and bone (Figure 2C,D). We investigated the effect of E2 treatment on the number of functional IL-17-producing cells in joints of mice with CIA and found that E2-treated mice had fewer IL-17-producing cells in their joints compared with mice administered placebo treatment (Figure 3A). In order to characterize the IL-17-producing cells in the joints, flow cytometry was used. An experiment terminated in the arthritis phase (day 35) showed that Th17 cell numbers were lower in joints of E2-treated mice (Figure 3B). Reduction in IL-17-producing cells was accompanied by decreased numbers of neutrophils in the joints of E2-treated mice (Figure 3C). Treatment with E2 inhibited arthritis development and was associated with decreased numbers of Th17 cells and neutrophils in joints.

### E2 increases the frequency of lymph node Th17 cells in early CIA

**Figure 4:**
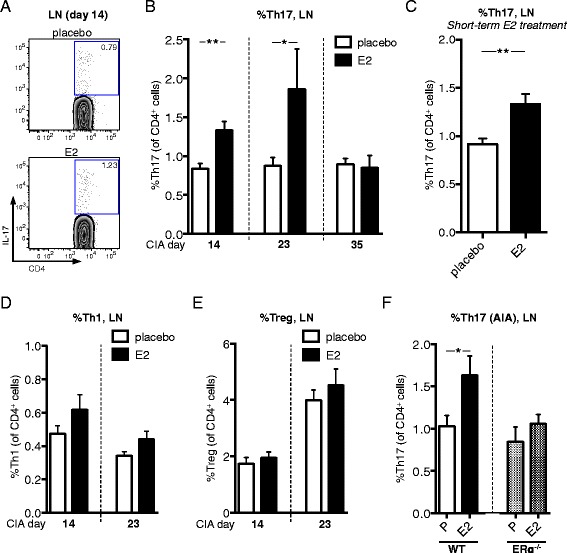
**Estrogenic effects on lymph node Th17 cells in collagen-induced arthritis and in antigen-induced arthritis.** DBA/1 mice were ovariectomized, subjected to collagen-induced arthritis (CIA) **(A–E)**, or ovariectomized estrogen receptor α–knockout mice (ERα^−/−^) mice and wild-type (WT) littermates were subjected to antigen-induced arthritis (AIA) **(F)** and treated with 17β-estradiol (E2; 0.83 μg/day) or placebo **(A, B)**. The frequency of T helper 17 (Th17) cells in lymph nodes (LNs) of CIA mice (terminated at day 14, *n* = 10 mice/group; at day 23, *n* = 8 to 10 mice/group; at day 35, *n* = 7 to 9 mice/group). Data from day 14 is from one representative experiment out of three independent experiments in total; otherwise, single experiments were performed. Representative flow cytometry plots are shown in **(A)**. **(C)** Frequency of Th17 cells in LNs of CIA mice (day 23, single experiment, *n* = 9 mice/group), with only 3 days of E2 treatment (days 20 to 22). **(D** **,** **E)** Frequencies of Th1 **(D)** and regulatory T cells (Treg) **(E)** in LNs of CIA mice (day 14, *n* = 10 mice/group; day 23, *n* = 8 to 10 mice/group; single experiments). **(F)** In AIA, frequency of Th17 cells in LNs (draining knee joints) was determined at day 14 (two independent experiments, in total *n* = 14 to 19 mice/group). To improve normal distribution of data, some data were log-transformed prior to statistical analysis (**B**, **D** and **E**). Arithmetic mean ± SEM is shown in all graphs. Student’s *t*-test was used (*P <0.05, **P <0.01).

In order to characterize the effects of E2 on Th17 cell development in arthritis, experiments were terminated at different time points during the induction phase of CIA (Figure 1), and Th17 cells in LNs were studied. Surprisingly, E2 increased the proportion of Th17 cells in LNs during the induction phase of CIA (days 14 and 23), whereas this effect disappeared in the arthritis phase (day 35) (Figure 4A,B). In order to investigate if E2 caused increased differentiation of Th17 cells in the LNs during the induction of CIA, or if the effect of E2 was more rapid, we performed an experiment with short-term E2 treatment. OVX mice were subjected to CIA as in the previous experiments, but they were treated with E2 for only 3 days (days 20 to 22), and the experiment was terminated on day 23. Interestingly, short-term E2 treatment also resulted in an increase of Th17 cells in the LNs, compared with controls (Figure 4C), clearly demonstrating that the effect of E2 on Th17 cells is rapid. Other populations of CD4^+^ T cells involved in development or regulation of arthritis, such as Th1 cells (Figure 4D) and Treg cells (Figure 4E), were unaffected by E2.

### E2-mediated increase in lymph node Th17 cells is also present in antigen-induced arthritis and is dependent on ERα

To verify that the effect of E2 on Th17 cells in arthritis was not specific for the CIA model, AIA was performed in placebo- or E2-treated OVX mice. We have recently shown that E2 inhibits AIA in an ERα-dependent manner [22]. In line with data on Th17 in the CIA model, E2 increased the frequency of Th17 cells in LNs of mice with AIA (Figure 4F, left). However, E2 did not change the frequency of LN Th17 cells in mice lacking ERα (Figure 4F, right).

### Absolute numbers of lymph node Th17 cells are increased by E2 in early CIA

**Figure 5:**
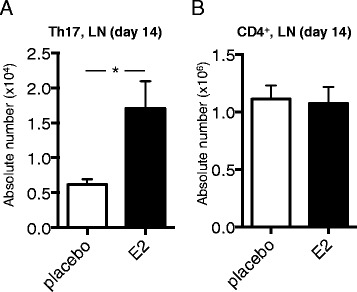
**Absolute numbers of lymph node CD4** ^**+**^ **cells and Th17 cells after estrogen treatment in early collagen-induced arthritis.** DBA/1 mice were ovariectomized, subjected to collagen-induced arthritis (CIA) and treated with 17β-estradiol (E2; 0.83 μg/day) or placebo. **(A,B)** Absolute number of T helper 17 (Th17) cells **(A)** and CD4^+^ cells **(B)** in one subiliac lymph node (LN) of CIA mice (day 14, *n* = 10 or 11 mice/group), obtained by multiplying cell population frequencies obtained by fluorescence-activated cell sorting by total LN cellularity. To improve normal distribution of data, some data were log-transformed prior to statistical analysis (A). Arithmetic mean ± SEM is shown in all graphs. Student’s *t*-test **(B)** or Welch’s *t*-test **(A)** was used (*P <0.05).

To further establish the E2-mediated increase in LN Th17 cells, absolute numbers of LN Th17 cells were analyzed. One subiliac LN was dissected from each mouse (CIA, day 14), and total numbers of lymphocytes were counted and related to frequency of Th17 cells (obtained by flow cytometry) to obtain absolute numbers of CD4^+^ cells and Th17 cells. In line with previous data on Th17 cell frequency in LNs (Figure 4B), the absolute numbers of LN Th17 cells were higher in mice treated with E2 compared with placebo (Figure 5A), whereas the total numbers of CD4^+^ cells were unaffected (Figure 5B).

### E2 influences lymph node chemokine production and chemokine receptor expression on Th17 cells

One explanatory mechanism for the increase of Th17 cells in the LNs during the induction phase of CIA could be that E2 interferes with the ability of Th17 cells to egress from LNs and migrate to joints. Therefore, we further characterized the effect of E2 on Th17 cell migration-associated pathways during the induction phase of CIA.

**Figure 6:**
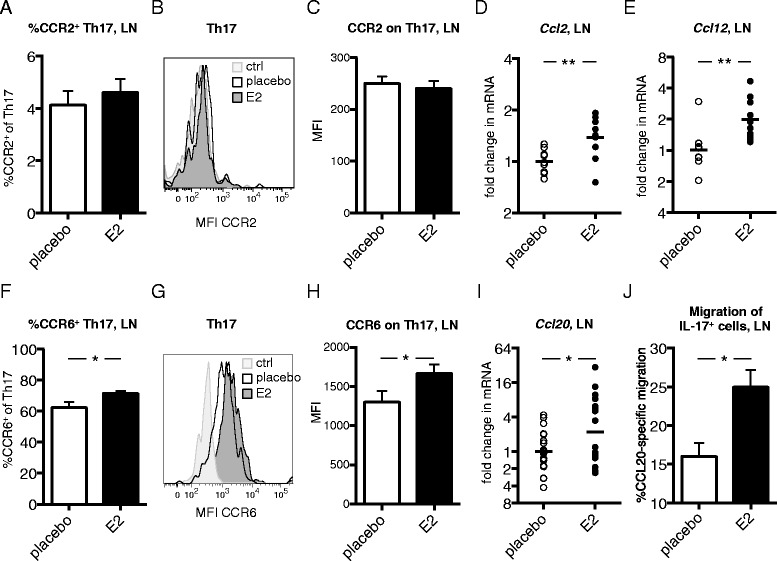
**Th17 chemokine receptor expression and corresponding lymph node chemokine expression after estrogen treatment in collagen-induced arthritis.** DBA/1 mice were ovariectomized, subjected to collagen-induced arthritis (CIA) and treated with 17β-estradiol (E2; 0.83 μg/day) or placebo. **(A–C)** Frequency of C-C chemokine receptor 2-positive (CCR2^+^) cells of T helper 17 (Th17) cells **(A)** and median fluorescence intensity (MFI) of CCR2 on Th17 cells **(B,C)** in lymph nodes (LNs) from CIA mice (day 14, single experiment with *n* = 10 or 11 mice/group). **(B)** Representative fluorescence-activated cell sorting (FACS) analysis plot where fluorescence minus one (FMO) is control. **(D,E)** mRNA expression of *Ccl2* **(D)** and *Ccl12* **(E)** in LNs of CIA mice (day 14, single experiment, *n* = 9 or 10 mice/group), determined by real-time quantitative PCR. **(F–H)** Frequency of CCR6^+^ cells of Th17 **(F)** and MFI of CCR6 on Th17 cells **(G,H)** in LNs of CIA mice (day 14, single experiments, *n* = 9 or 10 mice/group). **(G)** Representative FACS plot where FMO is control. **(I)** mRNA expression of *Ccl20* in LNs of CIA mice (day 14, data pooled from two experiments, *n* = 20 mice/group). **(J)** CCL20-specific migration of interleukin (IL)-17^+^ cells. LN cells from mice with CIA (day 35) were put on a transwell assay, with CCL20 in the lower chamber, and the frequency of migrated cells was evaluated with an IL-17 ELISPOT assay. Data are representative of two independent experiments (*n* = 3 mice/group). To improve normal distribution of data, some data were log-transformed prior to statistical analysis (**A**, **C**, **F** and **H**). Bars or lines show arithmetic mean ± SEM (**A**, **C**, **F**, **H** and **J**) or geometric mean (**D**, **E** and **I**). Student’s *t*-test (A, C–F, H and J) or analysis of covariance with experiment as covariate (I) was used (*P <0.05, **P <0.01).

A fraction of both human and murine Th17 cells express the chemokine receptor CCR2 that mediates migration toward CCL2 and CCL12 [29-31]. We confirmed expression of CCR2 on LN Th17 cells from mice with CIA terminated day 14; however, irrespective of treatment group, only a minor proportion (approximately 4.5%) of Th17 cells expressed CCR2 (Figure 6A). Moreover, the intensity of CCR2 expression on Th17 cells did not differ between the treatment groups (Figure 6B,C). We also analyzed mRNA levels of *Ccl2* and *Ccl12* in whole LNs and found that E2 increased *Ccl2* and *Ccl12* compared with placebo-treated controls (Figure 6D,E).

The interaction between CCR6 and CCL20 plays an important role in the migration of pathogenic Th17 cells in arthritis, and a majority of Th17 cells express CCR6 (Figure 6F) [19]. We analyzed CCR6 expression on LN Th17 cells from E2- and placebo-treated mice with early CIA (day 14). Interestingly, we found that E2 increased the proportion of CCR6^+^ cells in the Th17 population (Figure 6F), as well as enhanced the expression of CCR6 on Th17 cells, in comparison to the placebo group (Figure 6G,H). We considered the possibility that the egress of CCR6^+^ Th17 cells from the LNs could be hampered in E2-treated mice due to decreased production of CCL20 in joints and reduced serum levels of CCL20. However, serum CCL20 in mice with CIA terminated at day 14 did not differ between the treatment groups (mean ± SEM: placebo = 224.7 ± 16.13 vs. E2 = 212.4 ± 18.5 pg/ml; non-significant Student’s *t*-test), and CCL20 was not detectable in supernatants from joint cell cultures (CIA day 49). We then speculated that CCR6^+^ Th17 cells might be retained in the LNs because of increased levels of CCL20 within the LN tissue. Interestingly, we found increased *Ccl20* mRNA in LNs from E2-treated mice (Figure 6I) with CIA day 14, revealing one possible mechanism for E2-induced retention of Th17 cells during arthritis development.

In order to investigate if E2 could influence the function of IL-17-producing cells, LN cells from CIA mice (day 35) were used in an *in vitro* CCL20-induced chemotaxis assay. The results showed that IL-17-producing cells from E2-treated mice migrated better toward CCL20 than did cells from placebo-treated mice (Figure 6J), which implicates a functional relevance of the increased CCR6 expression on Th17 cells after E2 treatment, as shown in Figure 6H.

Naïve T cells generally downregulate the expression of CCR7 after activation to enable LN egress. We speculated that E2 might sustain the expression of CCR7 on Th17 cells and thereby hamper their egress from LNs. However, regardless of treatment, LN Th17 cells (CIA, day 14) did not express CCR7 at all (see Additional file 1: Figure S1A). As a positive control, CCR7 expression was confirmed on CD4^+^ cells, but was not altered by E2 treatment (see Additional file 1: Figure S1B–D). mRNA encoding the corresponding chemokines to CCR7, *Ccl19* and *Ccl21* was detected in whole LNs (CIA, day 14), and the results show that *Ccl19*, but not *Ccl21*, was reduced by E2 (see Additional file 1: Figure S1E,F).

### E2 modulates S1PR1 expression on Th17 cells in CIA

**Figure 7:**
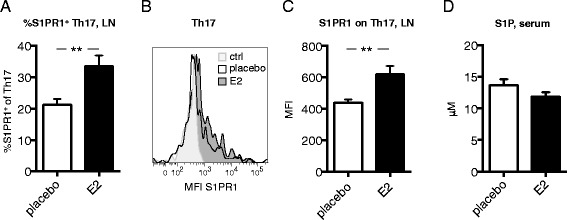
**Th17 S1PR1 expression in lymph nodes in early collagen-induced arthritis.** DBA/1 mice were ovariectomized, subjected to collagen-induced arthritis (CIA) and treated with 17β-estradiol (E2; 0.83 μg/day) or placebo. **(A–C)** Frequency of sphingosine-1-phosphate receptor 1-positive (S1PR1^+^) cells of T helper 17 (Th17) **(A)** and median fluorescence intensity (MFI) of S1PR1 on Th17 cells **(B,C)** in lymph nodes (LNs) from CIA mice (day 14, single experiment with *n* = 10 mice/group). **(B)** Representative fluorescence-activated cell sorting analysis plot where fluorescence minus one is used as a control. **(D)** S1P levels in serum of mice with CIA at day 14 were determined by enzyme-linked immunosorbent assay (single experiment, *n* = 9 or 10 mice/group). To improve normal distribution of data, some data were log-transformed prior to statistical analysis **(A,C)**. Bars or lines show arithmetic mean ± SEM. Student’s *t*-test (**P <0.01).

S1P and its receptor S1PR1 are important in mediating T cell egress from LNs [32]. The S1P gradient—with the lowest levels in the LN and the highest in lymph and blood—drives cell egress [33]. We hypothesized that the E2-induced retention of Th17 cells in the LNs could be caused by decreased expression of S1PR1 on Th17 cells or modulation of serum S1P levels. Unexpectedly, E2 increased the frequency of S1PR1^+^ Th17 cells, as well as the median fluorescence intensity of S1PR1 on Th17 cells (Figure 7A to C), in LNs of mice with CIA at day 14. Moreover, the levels of serum S1P at this time point did not differ between treatment groups (Figure 7D).

## Discussion

Our study shows that beneficial treatment with E2 in experimental arthritis is connected to a modulated distribution of Th17 cells whereby E2 increases Th17 cells in LNs and decreases Th17 in joints. In addition, we show that E2 alters the Th17 phenotype with regard to migratory function, as E2 increases CCR6 and S1PR1 on Th17 cells and also influences LN chemokine expression. We suggest that increased CCR6 on Th17 cells, as well as increased corresponding ligand (CCL20) within LNs, results in retention of Th17 cells in LNs during the induction phase of CIA. Probably, Th17 cell migration to joints is abrogated, which subsequently leads to decreased recruitment of neutrophils to the joints, reduced inflammation and alleviation of joint erosions.

The interaction between chemokines and chemokine receptors directs systemic T cell trafficking as well as localization of T cells within tissue microenvironments [34]. Migration of Th17 cells to the joints in arthritis is orchestrated mainly by CCL20 and corresponding receptor CCR6 expressed on Th17 cells [19]. E2 induces the expression of CCR1–CCR5 in murine splenic CD4^+^ T cells [35] and dose-dependently increases CCR6 in human lymphoblastoid cells [36]. In this study, we demonstrate that E2 upregulates the expression of CCR6 on Th17 cells (Figure 6G and H). Moreover, the E2-induced expression of CCR6 on Th17 cells is indeed functional, as cells from E2-treated CIA mice migrated better toward CCL20 *in vitro* compared with placebo-treated mice (Figure 6J). Probably, E2 directly affects transcription of CCR6 because the presence of two ER elements in the human *Ccr6* gene has been predicted by database studies [36], although this has not been studied in mice. Despite increased expression of CCR6 on LN Th17 cells, we found fewer Th17 cells in joints of E2-treated mice compared with placebo (Figure 3B). We report that E2 increases *Ccl20* mRNA in LNs (Figure 6I). CCL20 is produced predominantly in response to inflammation, by, for example, macrophages, dendritic cells and synovial fibroblasts [37]. We speculate that the increased *Ccl20* mRNA levels in LNs that we report could be explained by E2-mediated stimulation of CCL20 production from LN resident dendritic cells, macrophages or lymphoid stromal cells. Indeed, dendritic cells express ER, and estrogen has vast effects on these cells (reviewed in [38]). However, Th17 cells generated *in vitro* can also produce CCL20 [19]. To exclude the possibility that estrogen-mediated increase of *Ccl20* mRNA is solely an effect of increased Th17 cells, LN cells from mice (CIA day 14) treated with E2 or placebo were stimulated *in vitro* with plate-bound anti-CD3. Supernatants were collected after 2 days and assessed in CCL20 ELISA, but CCL20 was undetectable in all samples. Hence, we conclude that CCL20 in LNs is most likely produced by other cells than T cells.

We demonstrate that E2 increases the expression of mRNA encoding *Ccl2* and *Ccl12*, in addition to *Ccl20*, in the LNs (Figure 6D,E). These data are supported by a study showing that estrogen is a potent inducer of CCL2 and CCL12 secretion from mouse splenocytes [39]. We confirmed expression of CCR2 on Th17 cells; however, in accordance with a previous study [29], we could find expression of CCR2 only on a very small fraction of Th17 cells, and there was no difference between treatment groups (Figure 6A to C). Therefore, we conclude that the CCR2-CCL2/CCL12 pathway is not likely the major pathway responsible for E2-mediated retention of Th17 cells in LNs.

Another important pathway in T cell LN egress is S1P and its receptors (for example, S1PR1) [32]. Pharmacologic disruption of the S1P–S1PR1 interaction ameliorates CIA as a consequence of blocked lymphocyte egress from LNs [40]. Contradictorily, we report that E2—despite ameliorating CIA—increases S1PR1 expression on Th17 cells in early CIA (Figure 7A–C). We speculate that the enhanced expression of S1PR1 on Th17 cells after E2 treatment could be a result of decreased levels of S1P within the LNs of these mice, because high levels of S1P lead to internalization of S1PR1 [32]. However, according to the literature, levels of S1P within LNs are extremely low compared with lymph and blood, and therefore only S1P levels in serum were assessed [33]. Nonetheless, E2 did not influence serum levels of S1P (Figure 7D). Furthermore, the migratory function of S1PR1 could be modulated by other receptors, such as S1PR4 and CD69. Increased expression of S1PR4 and CD69 can, via different mechanisms, attenuate S1PR1-mediated chemotaxis of T cells [33,41,42]. Further, more detailed studies, including analysis of CD69 and other S1PRs, are therefore indispensable to fully understand the effects of estrogen on the S1P-S1PR system. Another possible explanation for the E2-mediated retention of Th17 cells, despite enhanced expression of S1PR1, could be that CCR6-CCL20 simply overrides the S1PR1-S1P-mediated migration of cells.

We cannot exclude that E2 might actually exert a stimulatory effect on the Th17 population in the induction phase of CIA. However, estrogen has been shown to regulate Th17 cells in various disease models [21,43]. Furthermore, our results demonstrating a decreased number of Th17 cells in the joints in the symptomatic phase of CIA (Figure 3B) do not favor a general Th17-inducing effect of E2. In addition, estrogen not only directly inhibits CIA but also delays the onset of disease, which is in accordance with our suggested model. The E2-induced Th17 cell increase in LNs in early CIA disappeared in the arthritic phase (Figure 4B, day 35). One possible explanation might be that Th17 cells retained in the LNs are undergoing apoptosis, possibly due to lack of signals required for the maintenance and pathogenicity of the Th17 lineage, such as IL-23 and transforming growth factor β3 [44].

All mice used in this study were OVX; hence, we can only speculate on how endogenous estrogens affect Th17 cells. However, the E2 dose used in the present study is considered to be physiologically relevant [26]. We have previously shown that endogenous estrogens are sufficient to reduce CIA, because OVX mice display more severe arthritis compared with sham-operated mice [45]. Moreover, it has been demonstrated that removal of estrogen by OVX results in modulation of Th17 cells in bone marrow and IL-17 levels in serum (in otherwise healthy mice), indicating that endogenous estrogens indeed regulate Th17 cells [43].

RA and multiple sclerosis are two diseases with many common features, such as a Th17-driven pathology, where estrogen treatment has been shown to be beneficial, both in experimental animal models and in humans [4,6,46]. Studies of experimental multiple sclerosis, EAE, have elucidated some mechanisms in the E2-mediated inhibition of the disease. Lelu *et al.* showed that ERα expression specifically in CD4^+^ T cells is required for the inhibiting effect of E2 in EAE and also that E2 reduces the frequency of splenic Th17 cells in an ERα-dependent manner [21]. In accordance, we have recently shown that the inhibitory effects of E2 on arthritis in CIA and AIA, and the associated bone loss in CIA, are clearly mediated via ERα [5,22]. In line with this, we report that the E2-induced Th17 cell increase in LNs is dependent on ERα (Figure 4F). Moreover, IL-17 has been ascribed a role as a mediator of bone loss induced by estrogen deprivation, which highlights the necessity of further studies in this field [43,47].

## Conclusions

In this study, we comprehensively characterized effects of estrogen on Th17 cells in experimental RA, demonstrating that estrogen regulates localization of Th17 cells during the development of arthritis, by increasing LN Th17 in early arthritis in an ERα-dependent manner and decreasing joint Th17 in established arthritis. We report that E2 influences Th17 migratory pathways in arthritis, suggesting that E2 treatment might result in Th17 cell retention in LNs thus preventing migration of Th17 cells to joints. Our study increases the understanding of the role of estrogen in autoimmune arthritis and opens up new fields for research concerning the sex bias in autoimmune disease.

## Additional file

Additional file 1: Figure S1. CCR7 expression and corresponding lymph node chemokine expression after estrogen treatment.

## Abbreviations

AIA: Antigen-induced arthritis
CCL: C-C chemokine ligand
CCR: C-C chemokine receptor
CIA: Collagen-induced arthritis
CII: Collagen type II
E2: 17β-estradiol
ELISA: Enzyme-linked immunosorbent assay
ERα: Estrogen receptor alpha
FMO: Fluorescence minus one
IFNγ: Interferon γ
LN: Lymph node
mBSA: Methylated bovine serum albumin
MFI: Median fluorescence intensity
OVX: Ovariectomy or ovariectomized
PBS: Phosphate-buffered saline
PMA: Phorbol 12-myristate 13-acetate
RA: Rheumatoid arthritis
S1P: Sphingosine-1-phosphate
SFC: Spot-forming cell
Th: T helper cell
Treg: Regulatory T cell
WT: Wild type
