# Supplementary material for: Estrogen regulates T helper 17 phenotype and localization in experimental autoimmune arthritis
Source: Arthritis Res Ther. 2015 Feb 13;17(1):32. doi: 10.1186/s13075-015-0548-y (PMC4355457; doi:10.1186/s13075-015-0548-y)
Supplement: Additional file 1: Figure S1. — CCR7 expression and corresponding lymph node chemokine expression after estrogen treatment. [file 13075_2015_548_MOESM1_ESM.pdf]

SUPPORTING INFORMATION

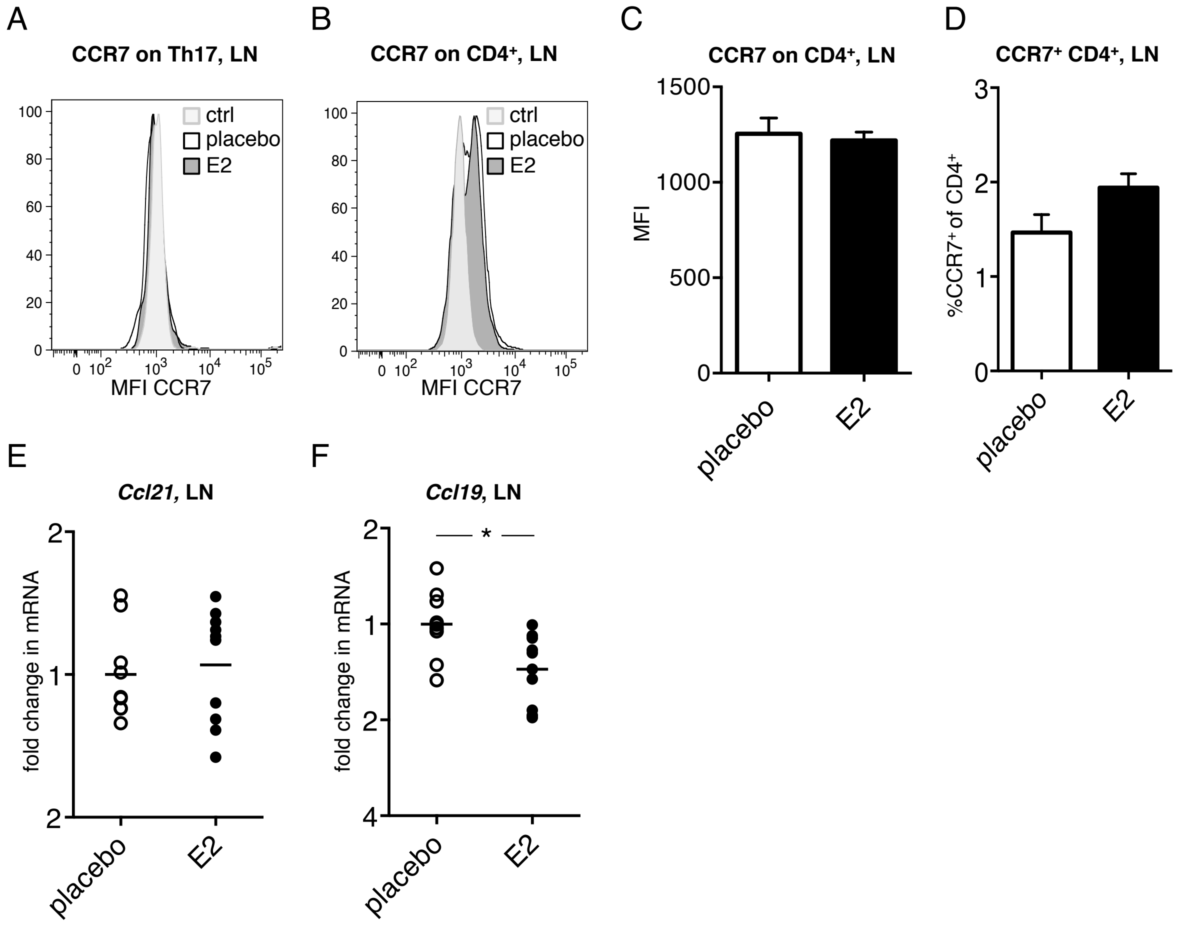

Figure S1. Expression of CCR7 and corresponding lymph node chemokine mRNA after estrogen treatment. DBA/1 mice were ovariectomized, subjected to CIA and treated with E2 (0.83 μg/day) or placebo. (**A-D**) FACS analysis of CCR7 on lymph node cells from mice with CIA day 14. Representative FACS plots where fluorescence minus one (FMO) is control, showing CCR7 expression on (A) Th17 and (B) all $CD4^+$ cells. (**C**) Median fluorescence intensity (MFI) of CCR7 on $CD4^+$ cells and (**D**) frequency of $CCR7^+$ cells of $CD4^+$ cells (single experiments, *n*=9-10 mice/group). (**E, F**) mRNA expression of chemokines in whole lymph nodes from mice with CIA day 14, (E) *Ccl21* and (F) *Ccl19* (single experiments, *n*=9-10 mice/group). Bars or lines show arithmetic mean + SEM (C, D) or geometric mean (E, F). Student's t-test (C, E, F) or Welch's t-test were used (D) (*p<0.05).
